# Xanthoma of rib: a case report and review of the literature

**DOI:** 10.1186/s13019-023-02315-0

**Published:** 2023-07-03

**Authors:** Emilia Mottola, Flavia Adotti, Angelina Pernazza, Carlo Della Rocca, Giulia D’Amati, Piergiorgio Nardis, Jacopo Vannucci, Massimiliano Bassi, Federico Venuta, Marco Anile

**Affiliations:** 1 Division of Thoracic Surgery and Lung Transplant, AOU Policlinico Umberto I, University of Rome Sapienza, Viale del Policlinico 155, 00161 Rome, Italy; 2 Department of Medico-Surgical Sciences and Biotechnologies, Polo Pontino-Sapienza University, Latina, Italy; 3 Department of Radiological, Oncological and Pathological Sciences, University of Rome Sapienza, Rome, Italy; 4 Interventional Radiology Section of Department of Radiological, Oncological, and Anatomopathological, Sciences of Policlinico Umberto I of Rome, Sapienza University of Rome, Rome, Italy

**Keywords:** Xanthoma, Rib, Hyperlipidemia, Thoracic trauma

## Abstract

**Background:**

Xanthomas are well-circumscribed benign proliferative lesions seen mainly in soft tissues. Usually, they are found in hyperlipidemia and familial hyperlipoproteinemia. Histologically, are characterized by macrophage-like mononuclear cells, multinucleated giant cells and abundant foam cells. The bone involvement, however, is notoriously rare and rib localization is extremely rare.

**Case presentation:**

A 55-year-old man performed a chest X-ray and a subsequent chest Computed Tomography scan showing a rib lesion that was surgically removed and a diagnosis of rib xanthoma was made. The patient presented an unknown condition of hyperlipidemia.

**Conclusion:**

Rib xanthoma can be discovered accidentally and can be helpful in identifying an unrecognized condition of hyperlipidemia.

## Background

Xanthomas (or Xanthofibromas) are well-circumscribed benign proliferative lesions seen mainly in soft tissues such as skin, tendons, fasciae and occasionally in the periosteum. On physical examination, they appear as semi-solid areas such as macules or papules or large nodules, generally yellow (Greek xanthos = yellow), due to the presence of carotene contained in the lipids [1].

Histologically, they are characterized by macrophage-like mononuclear cells, multinucleated giant cells, and abundant foam cells formed by macrophages because of the gradual intracellular accumulation of lipids captured by specific receptors or by the mechanism of phagocytosis [2].

Recently, their interest has increased for three reasons: the mechanisms involved in the development of these pathological lesions appear to be similar to those involved in the early stages of atherosclerotic plaques [3]; their development can signal a high risk of severe metabolic and cardiovascular diseases; they are useful diagnostic markers of severe hypertriglyceridaemia [4] and primary dysbetalipoproteinemia [5].

Bone xanthoma is rare [6] appearing mainly in the long bones of the appendicular skeleton, in male patients and over 20 years of age; in particular, rib localization is extremely rare. Therefore, we report a case of intraosseous rib xanthoma, propose a review of the literature and discuss clinical, radiological, histopathological aspects.

## Case presentation

**Fig. 1:**
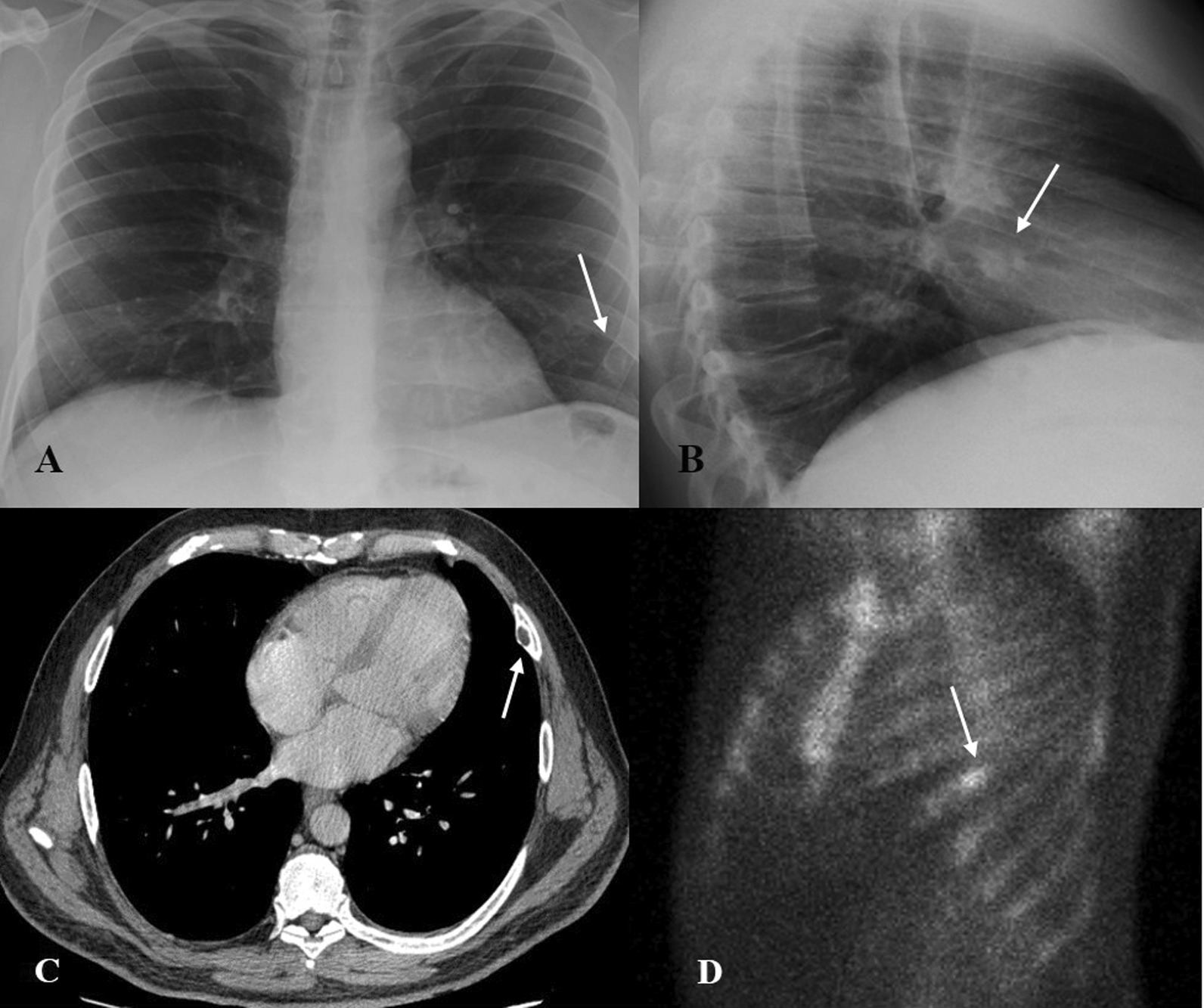
Preoperative imaging: **A** Chest X-ray shows an indefinite opacity in the left anterior arch of the 6th rib (white arrows), postero-anterior projection; **B** Latero-lateral projection of Chest X-ray; **C** CT scan showing a centimeter area with a central swelling and a surrounding sclerosis (white arrow) **D** An isolated hyperfixation of 6th rib is detected on 99m-tc HDP bone scan (white arrow)

A 55-year-old man with depressive disorders and grade I obesity performed clinical and radiological exams at the Department of Urology to undergo a biopsy of prostatic lesion, suspected for adenoma. A preoperative chest X-ray showed an indefinite opacity in the left anterior arch of the 6th rib (Fig. 1a, b). A chest Computed Tomography (CT) scan showed a centimeter area with a central swelling and a thick border of surrounding sclerosis (Fig. 1c).

In the suspicion of a primary bone tumor a 99m-technetium hydroxydiphosphonate (99m-Tc HDP) bone scan (Fig. 1d) was performed revealing an isolated hyperfixation of 6th rib.

The patient had no previous history of chest pain, trauma or cardiovascular disease. On physical examination, no further lesions appeared. Routine laboratory tests, including blood count and C-reactive protein, were normal.

**Fig. 2:**
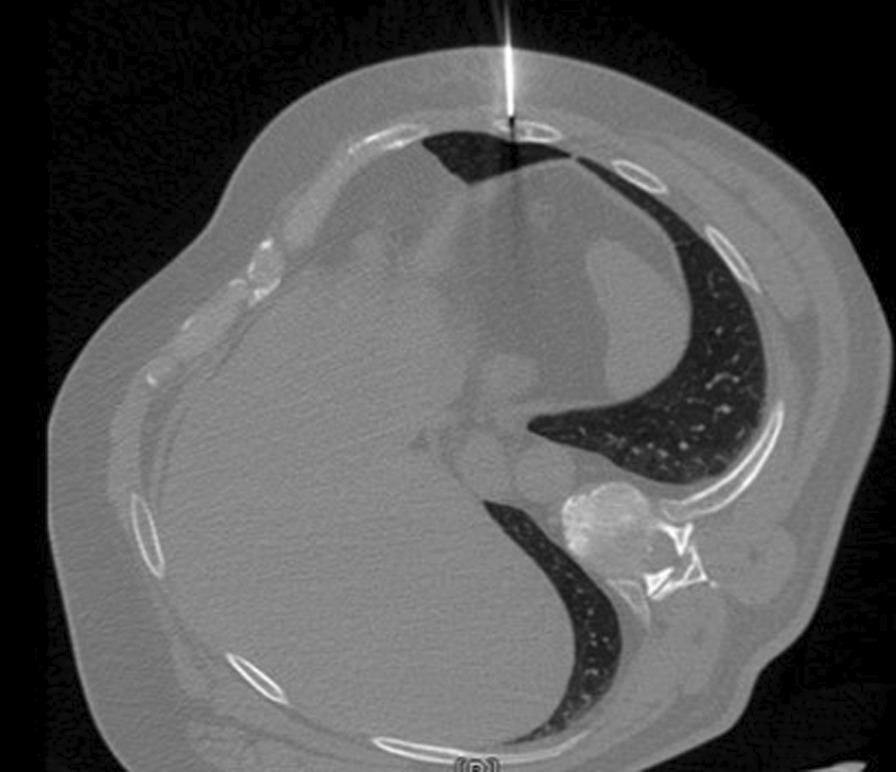
Percutaneous marker. Positioning of the percutaneous marker on the lesion under CT-guidance

After a multidisciplinary consultation with oncologists and radiotherapists and the placement of a CT-guided percutaneous marker (Fig. 2) the lesion was removed. Under general anesthesia, a two centimeters skin incision was made and limited exeresis of the underlying rib was performed, without any chest wall reconstruction. The postoperative course was uneventful and the patient was discharged the day after the operation.

**Fig. 3:**
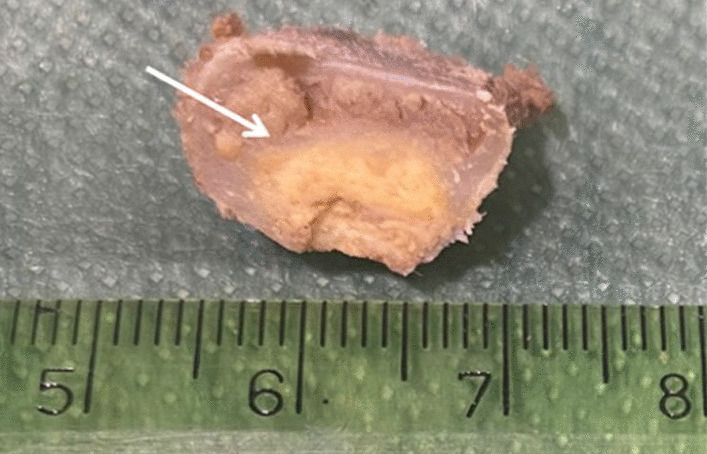
Macroscopic imaging. Rib’s cross section revealed a nodular, well definided, yellowish lesion, centered in bone marrow

**Fig. 4:**
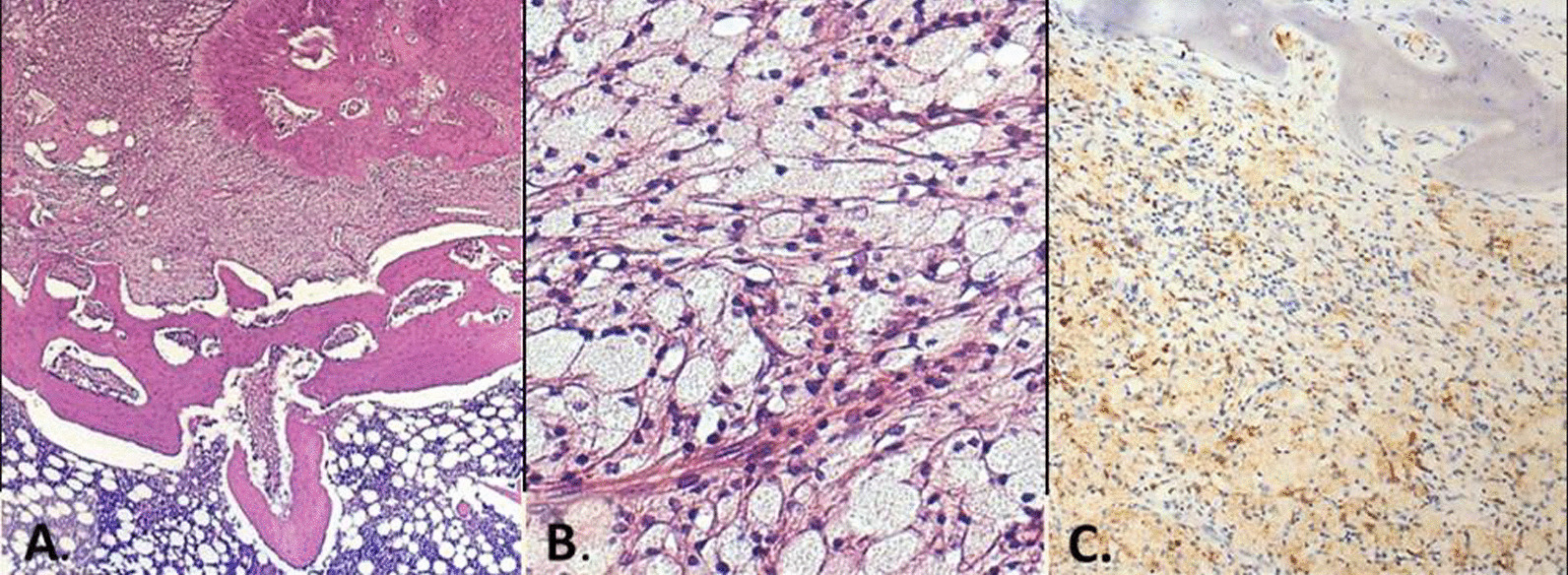
Histological examination. **A** Reactive bone with diffuse proliferation of foamy histiocytes (H&E, 4x); **B** dense foamy histiocytes aggregates with scant inflammatory cells (H&E, 20x); **C** diffuse positivity for CD68 (immunostaining, 4x)

On gross, a nodular, well defined, yellowish lesion, centred in bone marrow and measured cm 1, 5 was evident (Fig. 3). Histological examination revealed a proliferation of numerous foamy cells, multinucleated giant cells and occasionally Touton-type giant cells (Fig. 4a). Foamy cells were also intermixed with few spindle fibroblasts, lymphocytes, cholesterol clefts and fibrous tissue (Fig. 4b). The bone at the edge of the proliferation was diffusely reactive. Immunohistochemical analysis showed positivity for CD68 (Fig. 4c) and negativity for cytokeratin, PAX8, S100, FXIIIa, CD1a and BRAF V600E. Based on histological and immunohistochemical features diagnosis of Xanthoma of bone was made.

Due to the histological findings and suspecting an unknown hyperlipidemia, the lipid panel was performed showing hypercholesterolemia (345 mg/dl) and hypertriglyceridemia (589 mg/dl). The patient had never performed these laboratory tests before.

He also performed a color-Doppler Ultrasound of the supra-aortic vessels showing the presence of atherosclerotic plaques. He was referred to a family physician, began lipid-lowering therapy with statin drugs and a low-calorie diet. During the follow-up, the patient had lost weight, blood tests showed a reduction in lipid levels and no recurrences of xanthoma.

## Discussion and conclusions

Xanthomatous lesions of skin, blood vessels, abdominal viscera, and reticuloendothelial organs occur commonly as a complication of hyperlipidemic states. Mainly, they appear in male and obese patients in whom the lipids form deposits in soft tissue. Usually, they are found in hyperlipidemia and familial hyperlipoproteinemia, but only few have been seen in its absence [7].

The bone involvement, however, is notoriously rare. Generally, the most frequent localizations are the diaphysis of long bones, especially the tibia, but also the skull may also be included as mastoid air cells [8–10], mandibular bone [11], temporal [8–10] or frontal [12] bone which may cause cerebellar compression. The axial skeleton is not spared. A xanthoma case of sacrum [13] and calcaneus [14] has been described in the literature.

Xanthomas can be discovered accidentally or more commonly due to the appearance of pathological fractures and stinging pain. Sometimes patients may also have soft tissue involvement.

These tumors are described radiographically as sharply defined, lytic lesion with an expansible border that can often extend into the surrounding soft tissue [7], which is often responsible for bone deformities or the cause of pathological fractures [6].

Bone scintigraphy allows for mapping of the lesions and post-therapy follow-up [15]. Therefore, the radiological images remember other conditions, most commonly fibrous dysplasia, non-ossifying fibroma, cyst, and osteoblastoma [6]. For all these reasons, the diagnosis is histological. Generally, the lesions appear macroscopically as a localized and swollen area, with a soft consistency and yellowish, but they can also present as a cystic form with granular or fluid material. Microscopically, cholesterol vacuoles, giant cells, foam cells and fibrotic areas are present. The cytoplasm appears clear, vacuolated and with well-defined margins. Sometimes it is necessary to make a differential diagnosis with bone metastasis of clear cell carcinoma. Occasionally, spindle cells are presents, which has led investigators to include these lesions as a subset of benign fibrous histiocytoma (HBF) of bone. When HBF occurs as an inactive lesion in the metaphyseal portions of long bones, the complex is known as non-ossifying fibroma and be the end stage of a reparative phenomenon secondary to extensive bone resorption. Therefore, the differential diagnosis includes Erdheim-Chester disease [16], bone involvement of Rosai-Dorfman [17] disease and metastatic clear cell carcinoma. Finally, Xanthomas may represent a "burnt-out" benign condition such as fibrous dysplasia or histiocytosis X, in our case ruled out by anamnesis and laboratory data.

Based on the mode of onset and the contextual state of hyperlipidemia, it’s possible to classify bone xanthoma as reported:

- *Xanthomatous variant* xanthomatous changes in advanced stage of skeletal benign or malignant pre-existing lesions;
- *Secondary xanthoma* forms in the skeletal system of type-2 and 3 hyperlipidemic patients;
- *Primary xanthoma* with normal lipid metabolism

This classification may be helpful to allow diagnosis and treatment [18–21].

Xanthoma of rib is an exceptional localization and has been previously described in other nine cases as a condition frequently associated with thoracic trauma (Table 1).

**Table 1:** Summary of cases of rib xanthoma and their histopathological characteristics

References	Cases	Clinical history	Radiological finding	Pathology	Clinical aspects	Therapy
Bertoni et al. [6]	n. 3	History of trauma; Hyperlipidemia not mentioned	On CT-scan, all lesions are purely lucent. They appear as well-definited areas, sometimes expansile lytic lesion, with either a small area of surrounding reactive bone or a distinct sclerotic margin	The lesions are solid, soft, granular, and dull yellow Foam cells, giant cells, cholesterol clefts, and fibrosis are present in varying degree	Chest pain and soft tissue xanthomas	Surgical treatment
Lee et al. [22]	n.1	No history of trauma or hyperlipidemia	Tc-99m MDP bone images show an elongated main lesion in the left rib cage	Proliferation of foamy histyocites with areas of fibrosis and new bone formation along small vascular channel	Soreness over the left anterior thorax	Open biopsy
McDermott et al. [23]	n.3	Two cases with history of trauma Hyperlipidemia not mentioned	On CT-scan, the lesions vary from a small sclerotic focus to areas of lucency whit and whithout sclerotic margins Bone scan shows intense focal increase of radionuclide in single rib	Well-defined lesion in rib shows bright yellow central region surrounded by pale tan border. Dark red zone within lesion represents entrapped marrow Histollogically, they are relatively large, sheet-like core of histiocytes, having a pale, foamy to granular cytoplasm and small, round to oval hyperchromatic nuclei, admixed with small fascicles of bland-appearing spindle cells	One case with chest pain	Open biopsy
Blanco et al. [24]	n.1	History of minor trauma from an automobile accident No hyperlipidemia mentioned	The CT scan negative. Bone scan shows increased radionuclide uptake in one or more ribs and in the scapula	The external surface appears pink and focally hemorrhagic. On sectioning, the bone showed a tan yellow area Histologically, storiform patterns of spindle cells intermixed with lipid-laden histiocytes and giant cells are present	Asymptomatic	Open biopsy
Chon et al. [25]	n.1	No history of hyperlipidemia or trauma	On CT-scan, a 4-cm mass on fourth rib appears	Macroscopically, very suspicious for osteochondroma Histologically, appears a well-demarcated medullary lesion consisting of benign histiocytes and fibrous tissue	Chest pain and mucocutaneous lesions	Thoracoscopic rib resection

*CT* Computed Tomography, *Tc-99m MDP* 99m-technetium hydroxydiphosphonate

Bertoni et al. [6] had identified 21 cases of bone xanthoma between 1919 and 1988. Three cases of xanthoma of rib were present, associated with soft tissue xanthomas and pain in the rib cage. Lee et al. [22] reported the case of a 64-year-old man without a history of chest trauma and hypercholesterolemia but with chest pain . Another 3 cases were reported by McDermott et al. [23]. Blanco et al. [24] reported the case of a rib xanthoma secondary to thoracic trauma, in which the remodeling fibrous component prevailed over the lipid component . Recently, Chon et al. reported the case of a patient with rib xanthoma presenting with chest pain and mucocutaneous lesions without hyperlipidemia. This lesion was subjected to thoracoscopic rib resection due to the strong primary suspicion of an osteochondroma [25].

We have reported a case of Rib Xanthoma. However, it has some difference from the aforementioned cases. The patient had no history of chest pain or thoracic trauma. On physical examination, no other xanthomas were present. The radiological finding was very ambiguous, due to the prostatic lesion, and was suspected to be a bone metastasis. We decided to surgically remove the lesion with limited resection. After receiving the histological diagnosis, the patient performed a lipid panel which showed a hyperlipidemia’s disease. The patient had not performed these investigations for years and, due to depressive disorder and sedentary lifestyle, he had gained weight.

In conclusion, the xanthoma can represent an alarm signal of hyperlipidemia disease and atherosclerotic plaques of main vessels and its accidentally discovery can prevent the occurrence of cardiovascular disease.

## Data Availability

Not applicable.

## Abbreviations

CT: Computed tomography
Tc-99m MDP: 99M-technetium hydroxydiphosphonate
